# Genome-wide Prediction and Functional Validation of Promoter Motifs Regulating Gene Expression in Spore and Infection Stages of *Phytophthora infestans*

**DOI:** 10.1371/journal.ppat.1003182

**Published:** 2013-03-14

**Authors:** Sourav Roy, Meenakshi Kagda, Howard S. Judelson

**Affiliations:** Department of Plant Pathology and Microbiology, University of California, Riverside, California, United States of America; Oregon State University, United States of America

## Abstract

Most eukaryotic pathogens have complex life cycles in which gene expression networks orchestrate the formation of cells specialized for dissemination or host colonization. In the oomycete *Phytophthora infestans*, the potato late blight pathogen, major shifts in mRNA profiles during developmental transitions were identified using microarrays. We used those data with search algorithms to discover about 100 motifs that are over-represented in promoters of genes up-regulated in hyphae, sporangia, sporangia undergoing zoosporogenesis, swimming zoospores, or germinated cysts forming appressoria (infection structures). Most of the putative stage-specific transcription factor binding sites (TFBSs) thus identified had features typical of TFBSs such as position or orientation bias, palindromy, and conservation in related species. Each of six motifs tested in *P. infestans* transformants using the GUS reporter gene conferred the expected stage-specific expression pattern, and several were shown to bind nuclear proteins in gel-shift assays. Motifs linked to the appressoria-forming stage, including a functionally validated TFBS, were over-represented in promoters of genes encoding effectors and other pathogenesis-related proteins. To understand how promoter and genome architecture influence expression, we also mapped transcription patterns to the *P. infestans* genome assembly. Adjacent genes were not typically induced in the same stage, including genes transcribed in opposite directions from small intergenic regions, but co-regulated gene pairs occurred more than expected by random chance. These data help illuminate the processes regulating development and pathogenesis, and will enable future attempts to purify the cognate transcription factors.

## Author Summary

The genus *Phytophthora* includes over one hundred species of plant pathogens that have devastating effects worldwide in agriculture and natural environments. Its most notorious member is *P. infestans*, which causes the late blight diseases of potato and tomato. Their success as pathogens is dependent on the formation of specialized cells for plant-to-plant transmission and host infection, but little is known about how this is regulated. Recognizing that changes in gene expression drive the formation of these cell types, we used a computational approach to predict the sequences of about one hundred transcription factor binding sites associated with expression in either of five life stages, including several types of spores and infection structures. We then used a functional testing strategy to prove their biological activity by showing that the DNA motifs enabled the stage-specific expression of a transgene. Our work lays the groundwork for dissecting the molecular mechanisms that regulate life-stage transitions and pathogenesis in *Phytophthora.* A similar approach should be useful for other plant and animal pathogens.

## Introduction

Eukaryotic pathogens typically employ specialized structures for dissemination and infection. Most filamentous fungi and oomycetes, for example, proliferate in their hosts as vegetative hyphae, which generate spores that are used to reach new infection sites [1]. The spores of many plant pathogens, especially those with a biotrophic disease stage, germinate to form structures known as appressoria that are used to breach the host epidermis. Transitions between these stages requires the precise control of transcription, which is accomplished through interactions between transcription factors and their binding sites (TFBSs) in DNA [2]. Some transcription factors and their cognate TFBSs have been identified in filamentous fungal and oomycete pathogens [3]–[6], but relatively little is known about the structure or regulation of their promoters compared to those of model saprophytes and animals. Studies in *Saccharomyces cerevisiae* have shown that its promoters typically contain only a small number of regulatory sequences located a few hundred bases upstream of the transcription start site [7]. This contrasts with metazoans, where genes are also controlled by more distant motifs, which often bind many transcription factors and exert long-range effects across chromatin domains [8].

Identifying TFBSs in the promoters of pathogen genes is an important step towards characterizing the networks that regulate growth, differentiation, and pathogenesis. The classic strategy for identifying regulatory motifs by promoter mutagenesis is laborious and not suited to genome-wide application, especially in non-model systems which include most plant and animal pathogens. In recent years, bioinformatic analyses enabled by genome sequencing and expression profiling have helped accelerate the discovery of promoter motifs in model organisms. Typically, co-regulated promoters are searched for over-represented motifs using methods that include enumerative search, expectation maximization, or Gibbs Sampling algorithms [9]–[12]. Comparative genomics also offers methods for predicting motifs by searching cross-species promoter alignments for phylogenetic footprints, *i.e.* regions of conservation [13]. The over-representation and evolutionary approaches have both been used with some success, since many of the resulting motifs resemble those identified by traditional methods [10], [13]–[17].

Relatively little is known about the organization and function of promoters in oomycetes, a group of eukaryotes that includes important pathogens of plants and animals. Studies of the potato late blight pathogen *Phytophthora infestans* and relatives revealed a novel genome structure comprised of gene-dense and gene-sparse regions [18]. *P. infestans* grows by extending tubular hyphae which then form sporangia, each of which can release multiple biflagellated zoospores [19]. In response to external cues, the motile zoospores transform into walled cysts which extend germ tubes that form infection structures called appressoria. In prior studies, we used the traditional strategy of promoter mutagenesis to identify three motifs directing transcription during sporulation and zoosporogenesis [20]–[22].

Resources enabling high-throughput promoter analysis have recently been developed for *P. infestans*, including a genome sequence and microarray data [18], [23]. In this report, we combine bioinformatic and functional approaches to identify TFBSs involved in stage-specific expression. More than 100 motifs associated with five life-stages were identified based on over-representation analysis. Most are high-confidence candidates since they also showed conservation in related species or positional bias within promoters. Functional testing of six motifs using reporter genes in *P. infestans* transformants confirmed their predicted activities.

## Results

### Overview of transcriptional landscape in *P. infestans*

**Figure 1:**
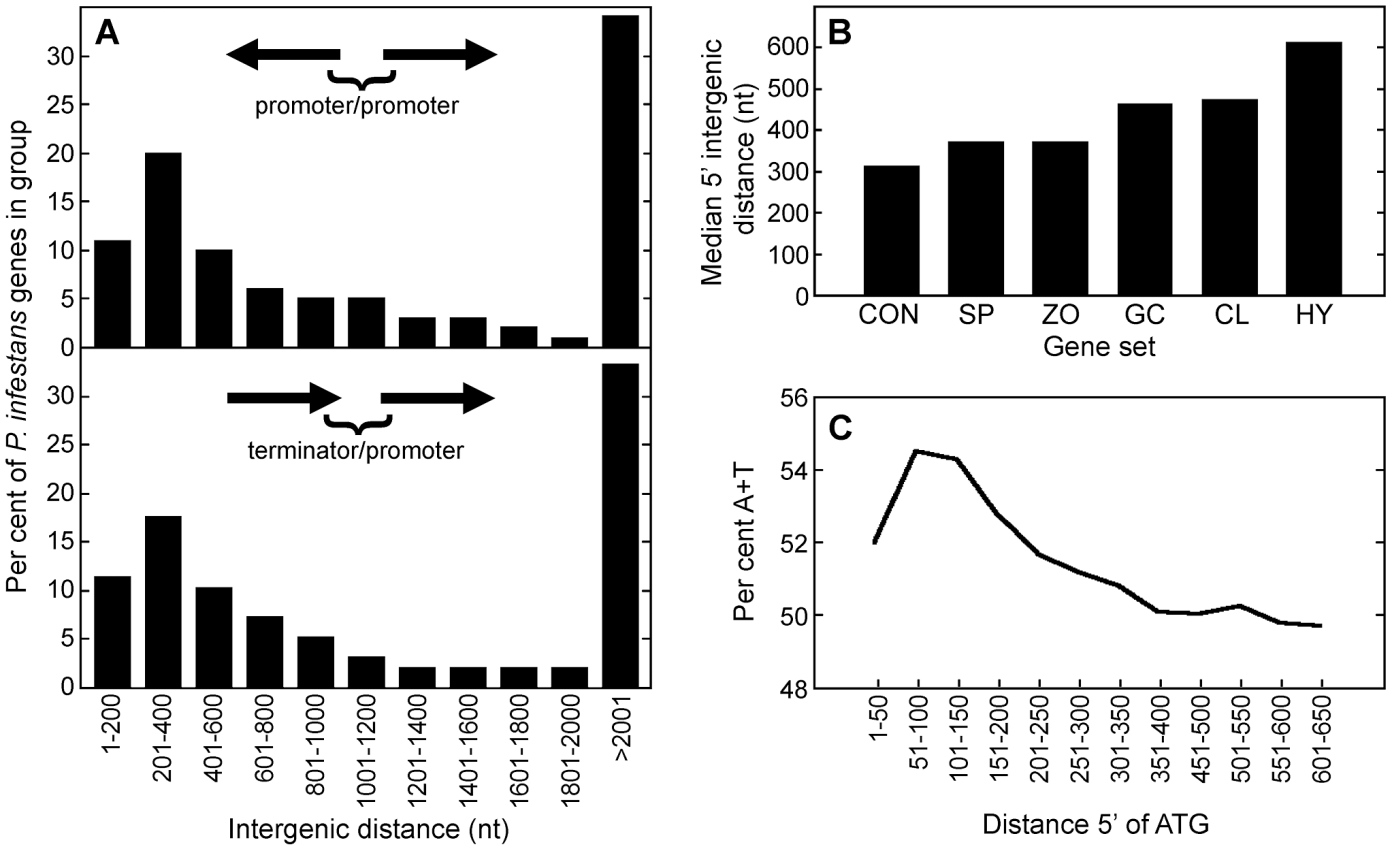
Characteristics of promoters in *P. infestans*. *(A)* Intergenic distances between genes oriented 5′ to 5′ and 3′ to 5′ are shown in the top and bottom graphs, respectively. Values are based on 15,471 genes from the largest *P. infestans* supercontigs. *(B)* Median 5′ intergenic distances in genes with different patterns of expression, based on the subset of genes having intergenic distances <2 kb. As described in Results, these were from constitutively expressed genes (CON), or those induced >7.5-fold in sporangia (SP), swimming zoospores (ZO), germinated cysts (GC), cleaving sporangia (CL), or hyphae (HY). *(C)* A+T content of DNA upstream of start codons, calculated in 50-nt bins. Little change was observed further upstream.

To obtain data for planning the strategy for motif discovery, the genome-wide distribution of intergenic distances, GC-content in intergenic regions, gene orientations, and stage-specific expression patterns were analyzed. Previous researchers reported that the *P. infestans* genome is partitioned into gene-dense and gene-sparse regions [18]. We repeated that analysis, incorporating data on gene orientation (Figure 1A). We focused on the 67% of genes that had 5′ intergenic distances of <2 kb, since their transcriptional regulatory sequences would be more likely to interact with those of adjacent genes. Of gene pairs separated by <2 kb, 41% are transcribed from a common intergenic region, with transcripts in the 5′ to 5′ orientation; in such cases the median intergenic distance was 430 nt. Since 5′ UTRs in *P. infestans* average about 41 nt [24], this implies that two functional promoters can reside within as little as 300 nt. By comparison, median intergenic regions in *S. cerevisiae*, *Arabidopsis thaliana*, and *Homo sapiens* are 0.45, 1.5 and 35 kb, respectively [25]–[27]. The remaining 59% of *P. infestans* genes are transcribed in the same orientation, with the 3′ end of one gene being adjacent to the 5′ end of its neighbor; their median intergenic distance was 441 nt.

We also studied if intergenic regions varied in size depending on how genes were expressed, as this would help indicate the best search space for motifs. This took advantage of a prior microarray study of five developmental stages [23]. Five sets of promoters from 100 genes induced strongly (>7.5-fold) in each of the stages were assembled. These were from genes up-regulated in sporangia compared to hyphae (“sporangia promoter set”), sporangia chilled for 1 hr to stimulate zoosporogenesis versus untreated sporangia (“cleavage set”), zoospores versus chilled sporangia (“zoospore set”), and germinating cysts forming appressoria versus zoospores (“germinating cyst/appressoria set”). A hyphal set was also developed from genes with higher mRNA levels in hyphae than the other stages. In addition, 150 constitutive genes were identified for which mRNAs varied by less than 25% between stages. Each gene model was curated manually, guided by EST data and sequences from *Phytophthora ramorum* and *Phytophthora sojae.* Corrections were applied to the 5′ ends of 13% of the *P. infestans* gene models.

The resulting data suggested that stage-induced promoters are larger than those from constitutive genes. This involved sorting the genes into groups with the different expression patterns, and then calculating median 5′ intergenic distances for the subset that were closely spaced, *i.e.* 2 kb or less from another gene (Figure 1B). Although each dataset spanned a broad range, the median 5′ intergenic region of constitutive genes was the smallest at 317 nt. Values from the inducible genes ranged from 373 for the sporangia set to 616 for the hyphal set. This resembles trends in other species, presumably since variably-expressed genes bind more transcription factors [25].

To develop background models for evaluating the statistical significance of motif frequencies, we also measured AT content 1-kb upstream of ATG codons. This averaged 49.6%, but rose to 54% near the start of genes (Figure 1C). The profile of the curve in the figure may reflect the small size of the typical *P. infestans* promoter, if its functional regions have a uniform AT content. Alternatively, the core promoter (the site that nucleates the assembly of a functional preinitiation complex; [28]) may be more AT-rich than other upstream regions, where most stage-specific TFBSs are expected. Genome-wide, intergenic regions are 49.3% AT compared to 46.1% for coding sequences [18].

### Influence of gene location and orientation on expression

In light of the close proximity of most *P. infestans* genes, we examined whether genes influenced the transcription of their neighbors. This would be relevant to motif discovery since a TFBS between two genes might influence both. For the analysis, we mapped expression patterns along *P. infestans* supercontigs and also calculated correlations between adjacent gene pairs.

**Figure 2:**
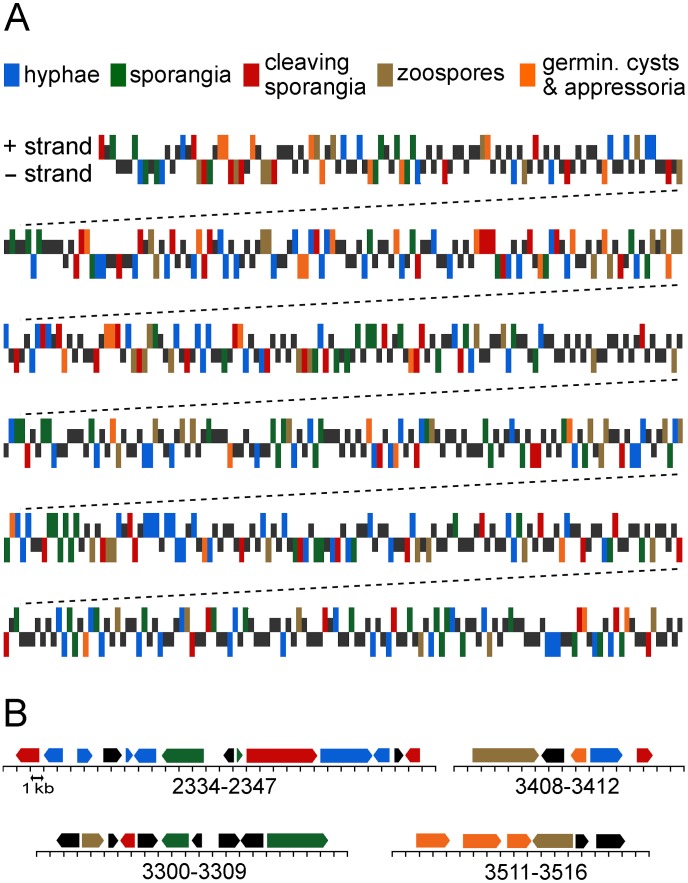
Genomics distribution of genes and expression patterns. *(A)* Expression profiles of genes within a representative region of a *P. infestans* supercontig. Genes with >2-fold higher mRNA levels in one life-stage compared to average are color-coded based when expression is highest, according to the key at the base of the figure. Genes not changing are marked in black, at half-height. The horizontal axis represents gene order and not distance, and marks above and below the axis represent genes transcribed to the right and left, respectively. *(B)* Four selected regions, drawn to scale, showing gene orientations and expression patterns using the key from panel A. One division equals 1-kb, and gene numbers trimmed of their PITG prefixes are shown below the scale.

For mapping expression patterns, we linked features in the microarrays to gene models in the *P. infestans* assembly, upon which the transcription patterns were plotted. This is illustrated in Figure 2A for a representative portion of Supercontig 1 (not drawn to scale), in Figure 2B for four selected regions (drawn to scale), and in Figure S1 for all genes. In these figures, genes showing >2-fold higher mRNA levels than average in one of the five stages are color-coded based on the stage with the maximum level; for example, green means highest in sporangia. Genes with the same stage-induced pattern were not typically adjacent, for example there were no physical clusters of genes having peak expression in sporangia. We also calculated the probability of adjacent genes having the same stage-induced pattern, focusing on 3744 pairs of neighboring genes as well as a subset of 2937 genes residing within 2 kb of each other; while *P. infestans* encodes about 17,797 genes, not all were represented or yielded signals on the arrays. With a few exceptions, adjacent genes showed unrelated patterns of stage-specific induction. Most exceptions involved tandemly repeated gene families, which would be expected to be co-expressed since both promoter and coding regions were likely to have undergone duplication. This occurred most for genes induced in the germinating cysts with appressoria stage; only for this stage were co-induced genes clustered more than expected by random chance at a 95% confidence interval. This was attributable to tandemly duplicated sets of β-glucanases, protein kinases, glucose transporters, and bZIP transcription factors, among others. One example is presented in the lower right portion of Figure 2B, which illustrates three co-expressed β-glucanases (PITG_03511, PITG_03512, and PITG_03513). A second example is an array of genes annotated as glucose transporters (PITG_13001 to PITG_13007). Such observations are consistent with prior studies that showed that genes induced in this pre-infection stage are rapidly evolving and prone to duplication [29].

In addition to the above which focused on the distribution of stage-induced patterns, we also measured global expression between gene pairs since this might detect subtle interactions. There was a weak tendency for pairs to be co-expressed, with an average Pearson correlation coefficient (*r*) of 0.11. Moreover, the distribution of *r* values between gene pairs and pairs from a scrambled dataset were significantly different based on a Kolmogorov-Smirnov test (*p*<0.001). The expression of 375 pairs were highly correlated (*r*>0.8) and 87 were anticorrelated (*r*<−0.8). Of the 375 co-regulated pairs, 53% were transcribed in the same direction, and 55% of these represented duplicated genes. In contrast, only about 10% of the co-regulated 5′-to-5′ genes were duplicated. There was little correlation between 5′ intergenic distance and co-regulation (*r* = −0.09).

### Discovery of over-represented stage-specific motifs

**Figure 3:**
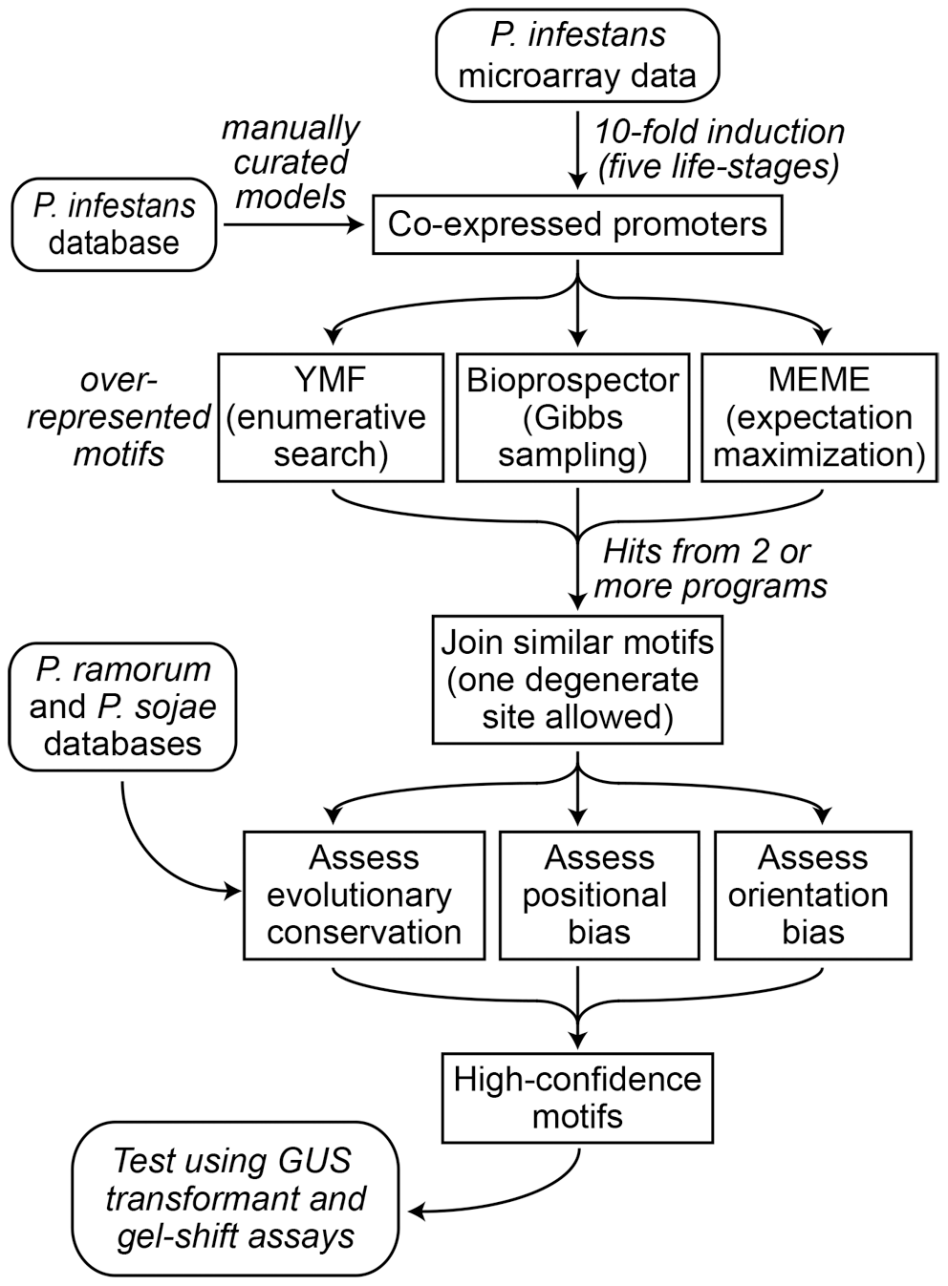
Strategy for characterizing stage-specific motifs from *P. infestans.*

The scheme illustrated in Figure 3 was used to identify candidate stage-specific TFBSs. In brief, the five sets of stage-induced promoters were searched for motifs that were over-represented compared to total *P. infestans* promoters. A search space of 1-kb of 5′ sequences was selected since this should include most TFBSs, based on the data in Figure 1B. The motifs were then tested for positional bias, orientation bias, and evolutionary conservation.

**Figure 4:**
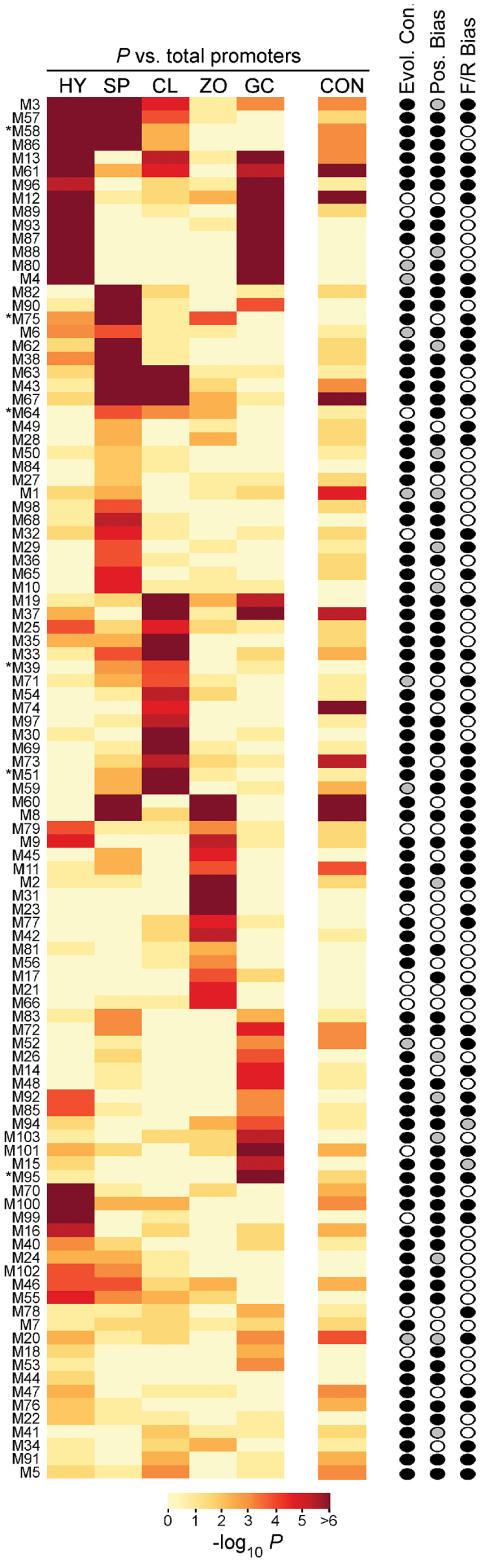
Features of the 103 motifs from *P. infestans*. The heat map indicates *p*-values associated with over-representation in the hyphal (HY), sporangia (SP), cleaving sporangia (CL), swimming zoospore (ZO), and germinated cyst (GC)-induced promoter sets compared to total promoters. Also graphed are *p*-values in constitutive promoters (CON). For clarity, *p*-values below 10^−6^ are shown as 10^−6^; the original values are in Table S1. The first column of ovals on the right of the heatmap indicates if motifs were evolutionarily conserved in *P. ramorum* or *P. sojae* (Evol. Con.), with black indicating yes in at least one species, white meaning no, and grey denoting ambiguous, *i.e.* motifs were found but in new locations. The next column indicates whether motifs were positionally biased (Pos. Bias), with black marking yes, white meaning no, and grey indicating ambiguous since the motifs did not occur enough in the relevant promoter database for trustworthy analysis. The column of ovals on the right indicates whether motifs had a significant (*p*<10^−2^) orientation bias, with black meaning yes and white no. An asterisk next to the motif name indicates those that were subjected to functional testing in *P. infestans* transformants.

Each of the five stage-induced datasets were searched separately for motifs using BioProspector, MEME, and YMF. These programs were selected since they employ independent methods and scored well in prior comparisons [11], [30]. We focused on promoters from genes induced >10-fold between developmental transitions (443 genes in the five promoter sets); this fold cut-off was raised compared to our earlier analyses to reduce noise in motif discovery. We also focused on motifs detected by at least two of the programs, allowing degeneracy at two sites. About 145 motifs fit this requirement, which were consolidated to 107 by joining those that were similar in sequence and had similar patterns of over-representation. Based on a *p*-value threshold of 10^−2^, 103 showed significant over-representation in at least one stage, which is shown in heat-map format in Figure 4; the motif sequences and number of hits in each dataset are in Table S1. The overall AT content of the 103 motifs was 49.9%, five were palindromes, and lengths ranged from 6 to 9 nt.

Approximately 80% of the 103 motifs were linked strongly to one developmental stage or two consecutive stages, and are therefore good candidates for binding sites for transcription factors that determine stage-specific expression. Based on the number of stages for each motif that passed the *p*-value threshold of 10^−2^, about 52 motifs were specific for a single stage. Examples include motif 82 (M82), which was significantly over-represented in sporangia-induced promoters (*p* = 10^−8^) but not the other sets, M95 which associated only with germinating cysts with appressoria (*p* = 10^−12^), and M99 which associated only with hyphae (*p* = 10^−12^).

About 21 motifs were significantly over-represented in promoters from two sequential stages, and 10 from three sequential stages. About half of the 21 were over-represented in the germinated cyst/appressoria and hyphal promoters, such as M87 (*p* = 10^−14^ and 10^−13^, respectively). This was not unexpected, since cyst germ tubes are very similar to hyphae and transition into hyphae. Several motifs were over-represented in sporangia and cleaving sporangia-induced promoters, such as M43 (*p* = 10^−11^ and 10^−7^, respectively). This was also not surprising since these stages are separated only by a 1-hr cold treatment, and many mRNAs induced in sporangia continue to rise during zoosporogenesis and/or during the zoospore stage. Accordingly, some motifs such as M64 were also over-represented in the sporangia, cleaving, and swimming zoospore promoters (*p* = 10^−4^ and 10^−3^, and 10^−3^ respectively). Likewise, several motifs were over-represented in hyphal and sporangia-induced promoters, such as M86 (*p* = 10^−6^ and 10^−8^, respectively). This may be explained by the fact that oomycete sporangia develop directly from hyphae, or that some tissue samples used for microarray analysis were not very synchronous. Regardless of the explanation, the approximately 80 motifs that associated with promoters from one or two sequential life stages are all good candidates for sites that bind transcription factors with stage-specific activities.

Two motifs matched the three promoter sites that were shown previously by mutagenesis to be needed for stage-specific transcription. M97, which was over-represented in the cleavage promoter set, is a close match (in the reverse orientation) to a site required for inducing the *NifC* gene during that stage [20]. Sporangia-associated motif M43 is an exact match to the site required for inducing the *Pks1* gene during sporulation [22], and a close match (in the reverse orientation) to the region needed to induce *Cdc14* during sporulation [21].

Not all motifs were associated only with consecutive stages. About 15 were over-represented in promoters from nonconsecutive stages, or both developmental or constitutive promoters (Figure 4, Table S1). One example is M60, which was over-represented in sporangia and swimming zoospore-induced promoters (*p* = 10^−16^ and 10^−13^, respectively) but not the intervening stage of cleaving sporangia (*p* = 0.8). A total of six motifs (M1, M8, M13, M60, M64, M67) occurred more in total promoters than expected by random chance; these may act as general enhancers.

### Positional and orientation bias

Many transcription factors need to act at a certain distance from the transcription start site or other regulatory locations, and therefore their TFBSs concentrate at a certain site within promoter space [31], [32]. Whether any of the motifs exhibited this bias was determined by mapping them within 200-nt bins from the relevant promoter set; 65 motifs were found to have positionally biased distributions (column “Pos. Bias” in Figure 4, Table S1). This may be an underestimate, since convincing evidence of bias could not be drawn for low-frequency motifs.

**Figure 5:**
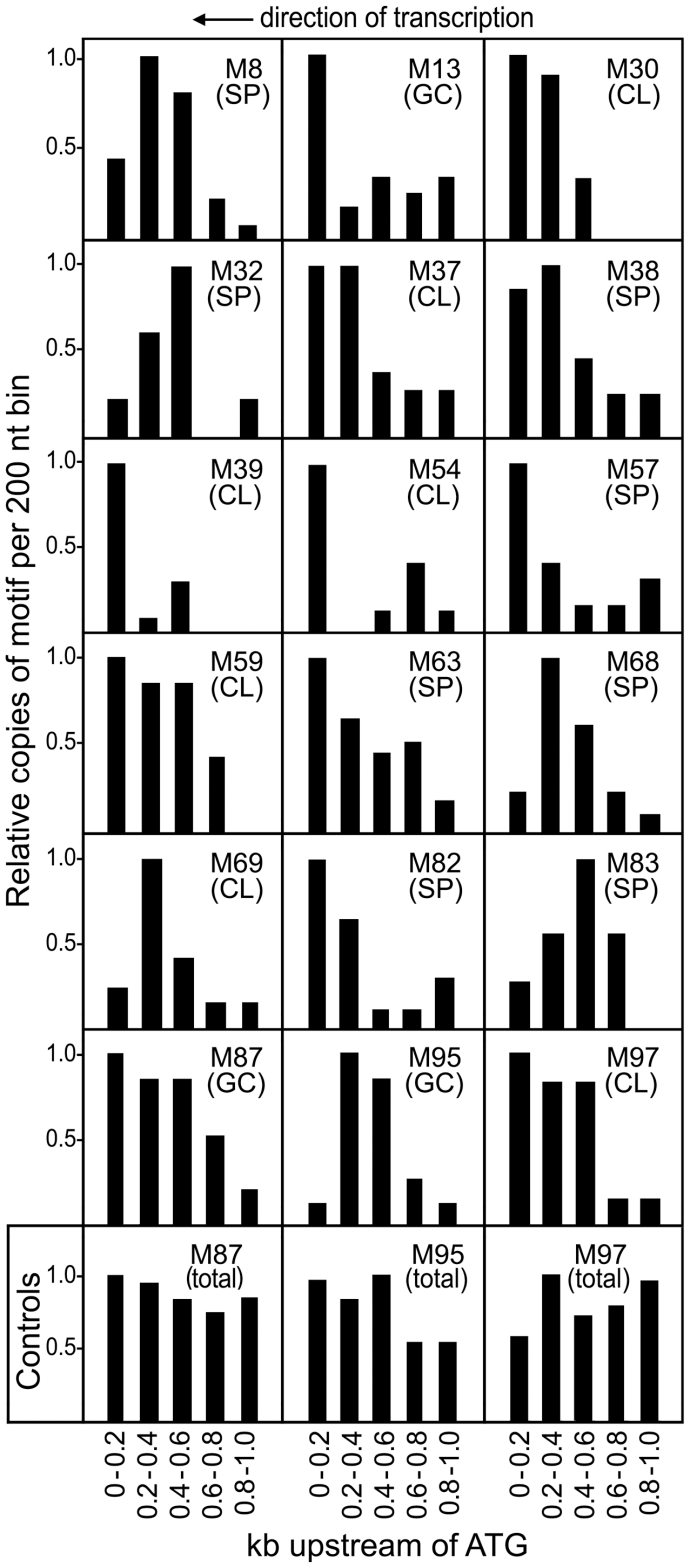
Positional bias of representative motifs within promoter space. The relative frequency of each motif is mapped across 200-nt bins taken from the relevant stage-induced promoter set, normalized to a value of 1.0 for the most populous bin. Marked in each panel is the motif and promoter dataset used for analysis, using the abbreviations in Figure 4. The bottom row shows three controls, indicating the frequency of three motifs in 2000 randomly selected promoters. The actual number of hits for each motif in the stage-induced promoter sets are in Table S1.

Data for 18 representative positionally biased motifs are shown in Figure 5. About one-third, such as M8 and M38, had distributions matching overall promoter size as shown in Figure 1A indicating that these TFBSs lack a strong positional bias. In contrast, motifs such as M68 and M83 tended to reside 200–600 nt upstream of the start codon. Others such as M30, M39, M53 (not shown in Figure 5), M57, and M82 were found closer to the transcription start site. M30 and M39 do not match known oomycete core promoter motifs, but M53 resembled the Inr or Initiator [33]. Interestingly, M53 was over-represented in promoters induced in the germinating cyst with appressoria stage.

As a control, we observed that similar biases were not observed in total promoters, where most matches may be false hits. This is illustrated at the base of Figure 5 for three representative motifs, M87, M95, and M97. These had biased distributions in induced promoters (Figure 5, second row from bottom), but very different patterns in total promoters (Figure 5, bottom row). Due to variation in AT-content across promoters (Figure 1C), the controls are not expected to have similar values in each bin. As AT-rich motifs, hits to M87 and M95 due to random chance are more common in the 3′ portion of total promoters, which are AT-rich. The opposite was observed for GC-rich motifs such as M97.

Some transcription factors must orient in a certain direction to fulfill their regulatory function. In *S. cerevisiae*, for example, 47% of TFBSs were found to have an orientation bias [32]. As shown in the column labeled “F/R bias” in Figure 4 and Table S1, this was the case for 50 of the 98 non-palindromic motifs (52%) from *P. infestans*, using a *p*-value threshold of 10^−2^. Cleaving sporangia-associated motif M33, for example, was detected 87 times in the forward orientation in the 95 cleavage-induced promoters but only 50 times in the reverse orientation. After correcting for the false discovery rate, the bias is even greater at 71 versus 27.

### Conservation of motifs in *Phytophthora*

**Figure 6:**
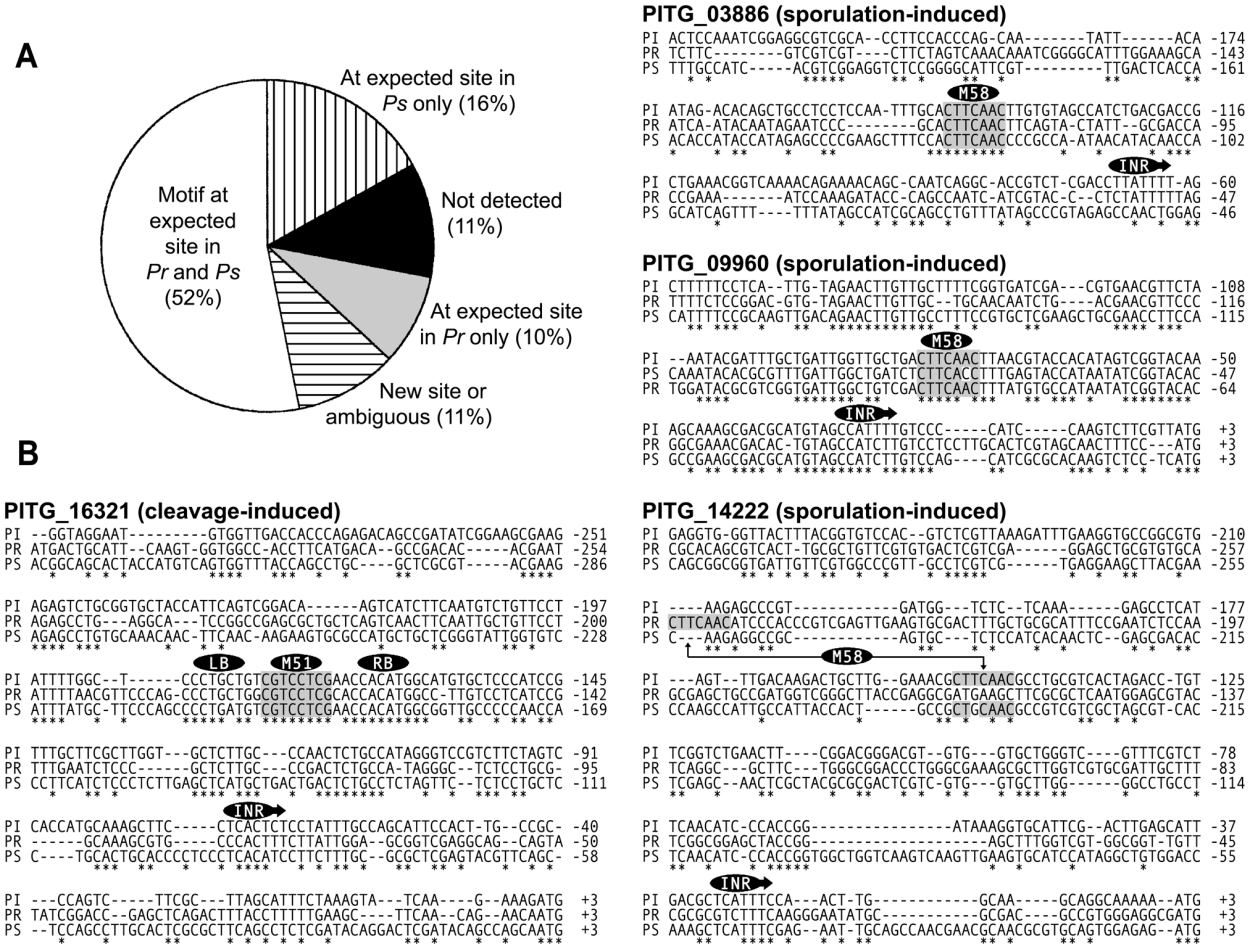
Interspecific conservation of motifs. *(A)* Pie chart illustrating the fraction of the 103 motifs that were detected at the same (*i.e.* expected) location in alignments of orthologous promoters from *P. infestans* (Pi), *P. ramorum* (Pr) and *P. sojae* (Ps), in the same location in *P. ramorum* or *P. sojae* compared to *P. infestans*, in a new location in *P. ramorum* or *P. sojae*, or not detected outside of *P. infestans. (B)* Representative CLUSTAL alignments. Shown on the left side of the figure are alignments of PITG_16321 (containing M51) with its orthologs from *P. ramorum* and *P. sojae.* Conserved blocks to the left and right of M51 are labeled as LB and RB, respectively. Shown in the right column are alignments of PITG_03886, PITG_09960, and PITG_14222, which contain M58. Also marked is the Initiator core promoter element, INR, which normally spans the transcriptional start site.

About 78% of *P. infestans* motif candidates were judged to be conserved in orthologous promoters from *P. ramorum* or *P. sojae*. A conclusion about whether a motif was conserved was developed by aligning promoters from about five genes; a match in at least some was considered to be indicative of conservation. Assessments for each motif are shown individually in the “Evol. Con.” column in Figure 4 and in Table S1, and results for all motifs are summarized in Figure 6A. Evidence for conservation between *P. infestans* and both of the other two species was obtained for 52% of motifs. About 10% of motifs were conserved between *P. infestans* and *P. ramorum* only, and 16% between *P. infestans* and *P. sojae* only. In 11% of cases, motifs were absent from both *P. ramorum* and *P. sojae*. In 11% of cases, the motifs were detected at new locations in one or both species; this was taken as an ambiguous result, since while promoter rearrangements are common [34] they are hard to distinguish from false hits.

Figure 6 shows representative alignments where conservation was detected. For cleavage-induced *P. infestans* gene PITG_16321 and its *P. ramorum* and *P. sojae* orthologs, for example, perfect matches to M51 were detected in the same location in all three species. The PITG_16321 alignment also reflects the common relationship seen between orthologous promoters: two to four sequence blocks are typically conserved. One usually spans the transcription start site, which in this case contains an Initiator-like sequence at −71 in *P. infestans*. Other conserved regions are typically found 40–200 nt upstream. For PITG_16321 these are the M51-containing block at −177, and another at −114. As will be shown later, the −114 block and conserved nucleotides a few bases to the left and right of M51 do not determine stage-specific expression.

Results for three ortholog sets containing sporulation-associated motif M58 are also shown in Figure 6. For PITG_03886, M58 is conserved perfectly in the three species. In PITG_09960, a three-way match also exists allowing for one base change in *P. sojae;* this was scored as a positive hit, since TFBSs often vary between species [35]. The PITG_14222 alignments show M58 at the same location in *P. infestans* and *P. sojae*, but 80-nt upstream in *P. ramorum.* Since the latter could be a false hit, our scoring scheme classifies M58 in PITG_14222 as conserved only between *P. infestans* and *P. sojae.* Since M58 was at the expected location in *P. ramorum* and *P. sojae* orthologs of PITG_03886 and PITG_09960, however, its overall classification is “conserved”.

### High-confidence motifs

**Figure 7:**
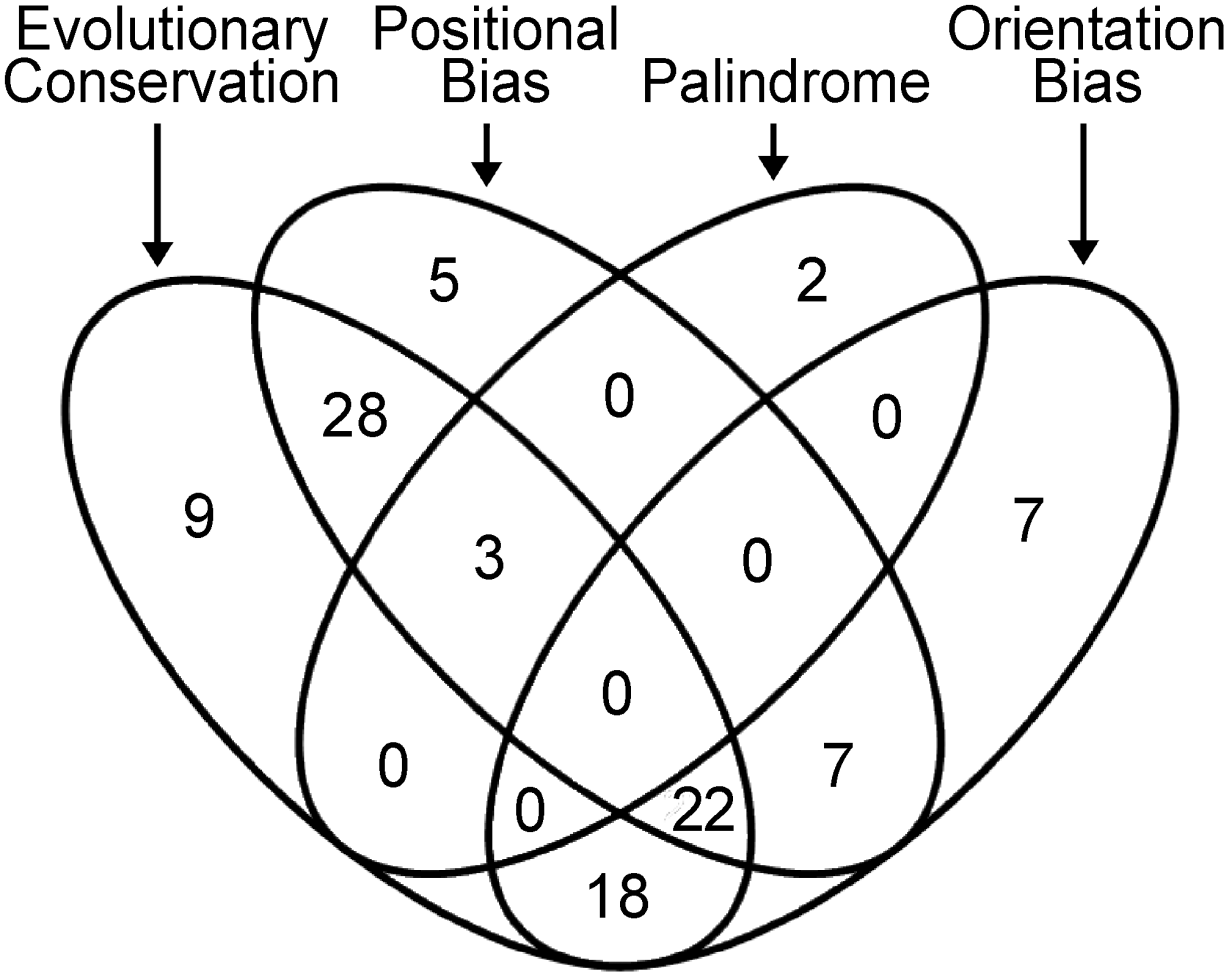
Summary of results from analysis of evolutionary conservation, positional bias, forward/reverse directionality, and palindrome analysis. Of the 103 predicted TFBSs, 101 show at least one of these features and are included in the figure.

Nearly all of the motifs demonstrated one or more characteristics typical of authentic TFBSs besides over-representation, such as interspecific conservation, positional bias, orientation bias, or palindromy (Figure 7). Of the 103 motifs, 101 had at least one of these features in addition to over-representation, 78 had at least two, and 25 had three. These classifications help indicate which motifs have the highest likelihood of having a function, in addition to suggesting how they interact with the transcriptional apparatus.

The two motifs lacking these additional characteristics were M1 and M66. These may still be real TFBSs, since not all experimentally confirmed sites exhibit positional bias or directionality, or reside in the same location in orthologs. The two motifs were over-represented in at least one developmental stage with *p*-values ranging from 10^−4^ to 10^−5^ and thus are unlikely to be false hits.

A few of the “high-confidence” motifs were close in sequence. These were M93 (TACATGTA) and M94 (TACCGGTA), which are palindromes differing only at the two central bases, M32 (AGC[AG]CAAG) and M34 (AGCTGAAG) which also differ at the two central bases, and M16 (AAATAAA) and M91 (TAAATAA) which overlap. As mentioned earlier, most motifs from BioProspector, MEME, and YMF had been merged if they differed at two or fewer sites and were over-represented in the same stages. The six motifs remained unmerged since their biases and/or probability distributions and varied. For example, M16 but not M91 was over-represented in hyphal-induced promoters (*p* = 10^−6^ and *p* = 10^−1^, respectively), and M93 was more over-represented than M94 in hyphal promoters (*p* = 10^−26^, *p* = 10^−2^).

### Functional tests of motifs

Six of the motifs were subjected to experimental analysis to see if they could drive β-glucuronidase (GUS) expression with the expected stage-induced pattern in transformants of *P. infestans*. As described below, each yielded the expected pattern. First analyzed was M51, which was predicted to confer expression during zoosporogenesis (*i.e.* sporangial cleavage). Interestingly, M51 is flanked by two sequence blocks that are conserved in *P. ramorum* and *P. sojae*, which are labeled LB and RB in Figure 6. These flanking sequences were not over-represented in cleaving sporangia promoters, but we considered the possibility that our definition of M51 was smaller than the authentic TFBS.

**Figure 8:**
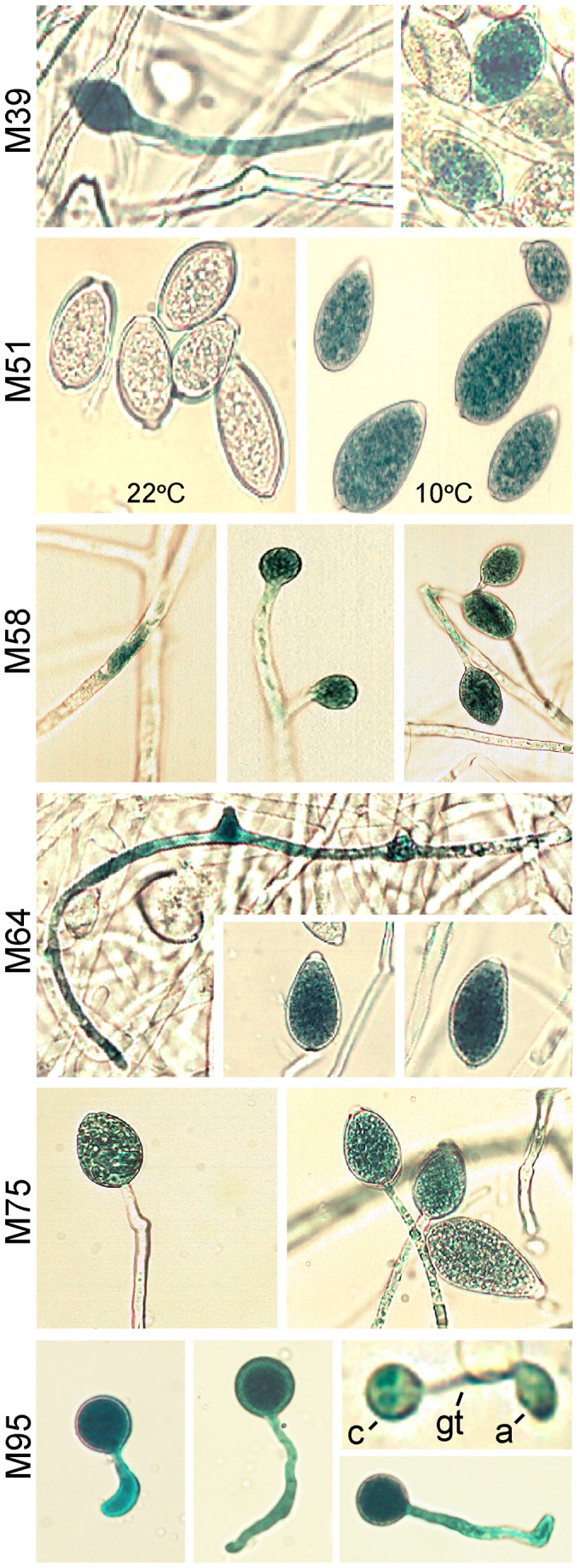
Histochemical staining of GUS under control of stage-specific motifs in stable transformants of *P. infestans*. For cleavage-associated motif M51, shown are the staining patterns of sporangia kept at 22°C or incubated at 10°C for 1-hr, in a representative transformant containing pDEL187. No staining was seen in hyphae. Similar patterns were seen in transformants in which M51 alone was fused to the *NifS* minimal promoter (not shown). For the other motifs, the transformants contained fusions of the motifs to the minimal promoter, and the images presented illustrated when GUS expression was first detected. These were at early and late stages of sporulation (M39, M58, M64, M75) or in germinated cysts (M95). Labeled in the M95 panel are the cyst (c), germ tube (gt), and appressorium (a).

**Figure 9:**
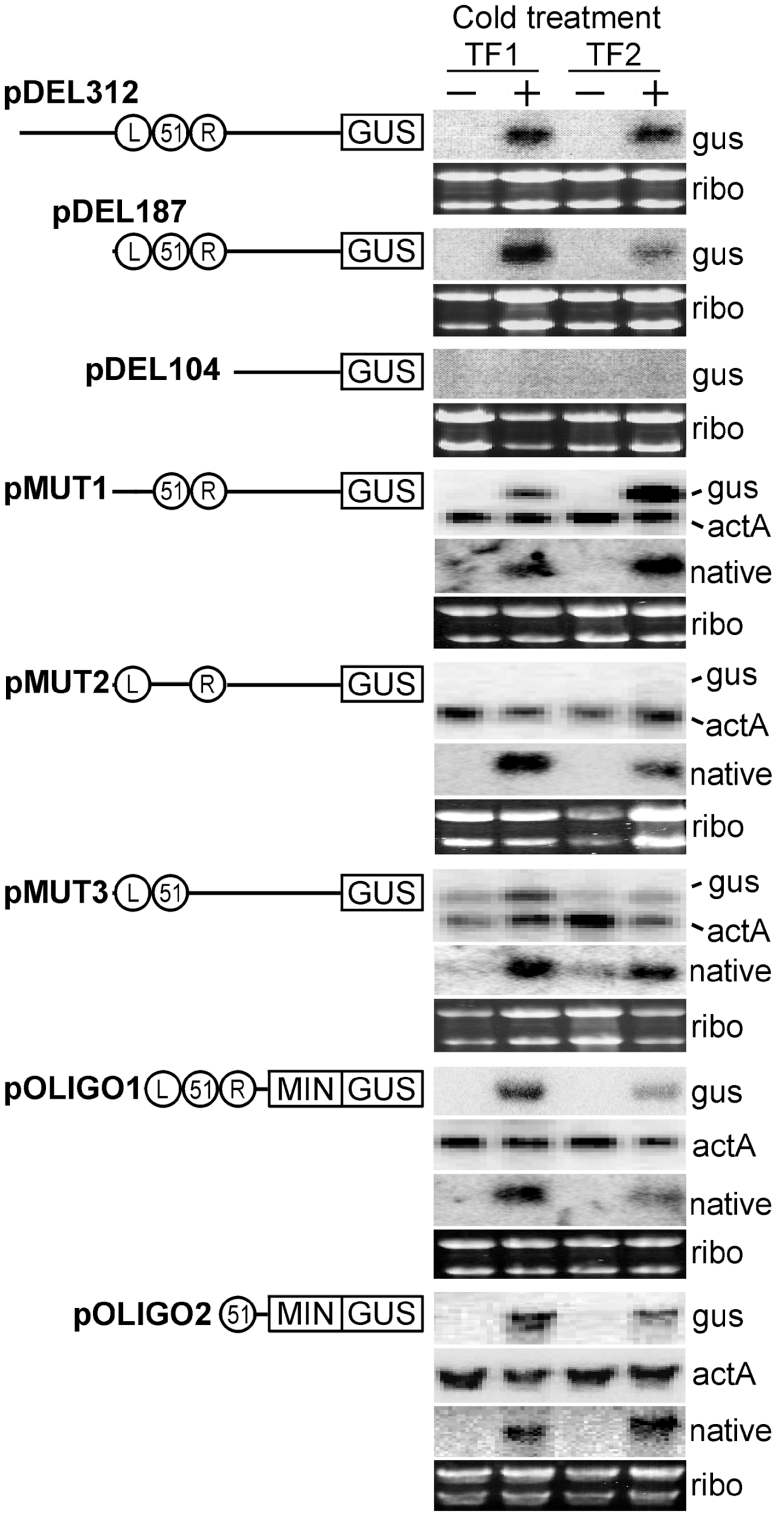
Constructs used to demonstrate function of M51 from promoter PITG_16321. Each contains sequences fused to the GUS reporter, which was transformed into *P. infestans*. Shown for each construct are results from RNA blot analysis of two representative transformants (TF1, TF2). RNA was extracted from sporangia immediately after harvesting (−) or after a 1-hr cold treatment (+). RNA was hybridized with probes for the GUS reporter (gus), or the PITG_16321 open reading frame to detect the endogenous transcript (native). The top three constructs contained serial 5′ deletions of the wild-type promoter, and the bottom five contained oligonucleotides with the LB, M51, and RB sequences (represented by circles) fused to the minimal *NifS* promoter (MIN) and GUS. Shown as loading controls are a photograph of rRNA and hybridization with the *actA* (actin) gene of *P. infestans.*

Initial experiments showed that at least part of the LB-M51-RB region was required for zoosporogenesis-specific expression. Plasmid pDEL312, which contains a 312 nt promoter fused to GUS, yielded expression in sporangia treated at 10°C for 1-hr to induce the cleavage of sporangia into zoospores, but not sporangia maintained at 22°C; for this plasmid and others described below, similar results were observed in multiple transformants. The zoosporogenesis-specific activity of the promoter fragment was shown first by histochemical staining (as in Figure 8), and later by RNA blot analysis in which bands of the expected size were detected only in the chilled samples (Figure 9). No activity was seen in hyphae. Indistinguishable results were obtained using a 500 nt promoter (not shown). pDEL187, which lacked bases upstream of the LB-M51-RB region, showed the same staining pattern and gene induction was confirmed by RNA blot analysis. pDEL104, which lacks the LB-M51-RB block, showed no expression.

Subsequent experiments specifically tested the functions of LB, M51, and RB by mutating those regions within pDEL187, and led to the conclusion that only M51 conferred stage-specific expression. Similar results were obtained from histochemical staining (not shown) and RNA blot analysis (Figure 9). Specifically mutating LB had no effect on stage-specific expression (pMUT1), while altering M51 prevented expression (pMUT2). As a control, we showed that the native gene in the transformants was induced in sporangia by the cold-treatment. Mutating RB did not block cold-induction of the transgene, although its basal expression seemed to be slightly elevated (pMUT3). Next, oligonucleotides containing the LB-M51-RB block or M51 alone were fused to the *NifS* minimal promoter, which contains a transcriptional start site but is not expressed on its own [21]. As shown in Figure 9, a fusion of LB-M51-RB to the minimal promoter, separated by a 37-nt spacer of random DNA, drove the normal chilling-specific expression of GUS (pOLIGO1). As a final and definitive test, an oligonucleotide containing M51 alone was shown to also confer this wild-type pattern to transformants (pOLIGO2).

Since the above experiments indicated that at least some motifs could act autonomously, we next tested five other predicted stage-specific motifs by fusing them one at a time to the *NifS* minimal promoter. Motifs M39, M58, M64, and M75 were most over-represented in the sporangia stage, and each resulted in the sporulation-specific accumulation of GUS (Figure 8). No staining was seen in nonsporulating hyphae. The effects of the motifs were subtly different, however. Transformants containing M58 showed GUS staining at the earliest stage; these showed expression within hyphae soon after cultures were stimulated to sporulate, and then later in sporangiophore and mature sporangia. This illustrated in Figure 8 where the three panels show (left to right) staining within a small segment of a hypha in a sporulating culture, immature sporangia (lacking basal septa and papilla), and mature sporangia. Transformants containing M39 and M64 first exhibited GUS staining in hyphal-like structures that are presumed to be sporangiophores, and then in mature sporangia. In contrast, expression driven by M75 seemed to be activated at a later stage, since staining was first observed in sporangia near maturity. It should be noted that while M58 was most over-represented in the sporangia stage (*p* = 10^−8^), it was also over-represented in hyphae (*p* = 10^−6^) and constitutive promoters (*p* = 10^−3^); perhaps it binds a transcription factor which does not become activated until sporulation is induced.

Also tested as a fusion with the minimal promoter was M95, which was associated with transcription in germinated cysts and appressoria. This resulted in the accumulation of GUS in germinated cysts, including their germ tubes and appressoria (Figure 8). Staining was first observed 2 hours after encystment. No expression was observed in hyphae, sporangia, chilled sporangia, or zoospores. As described later, this motif is associated with the expression of many pathogenesis-related proteins.

### Gel-shift assays

**Figure 10:**
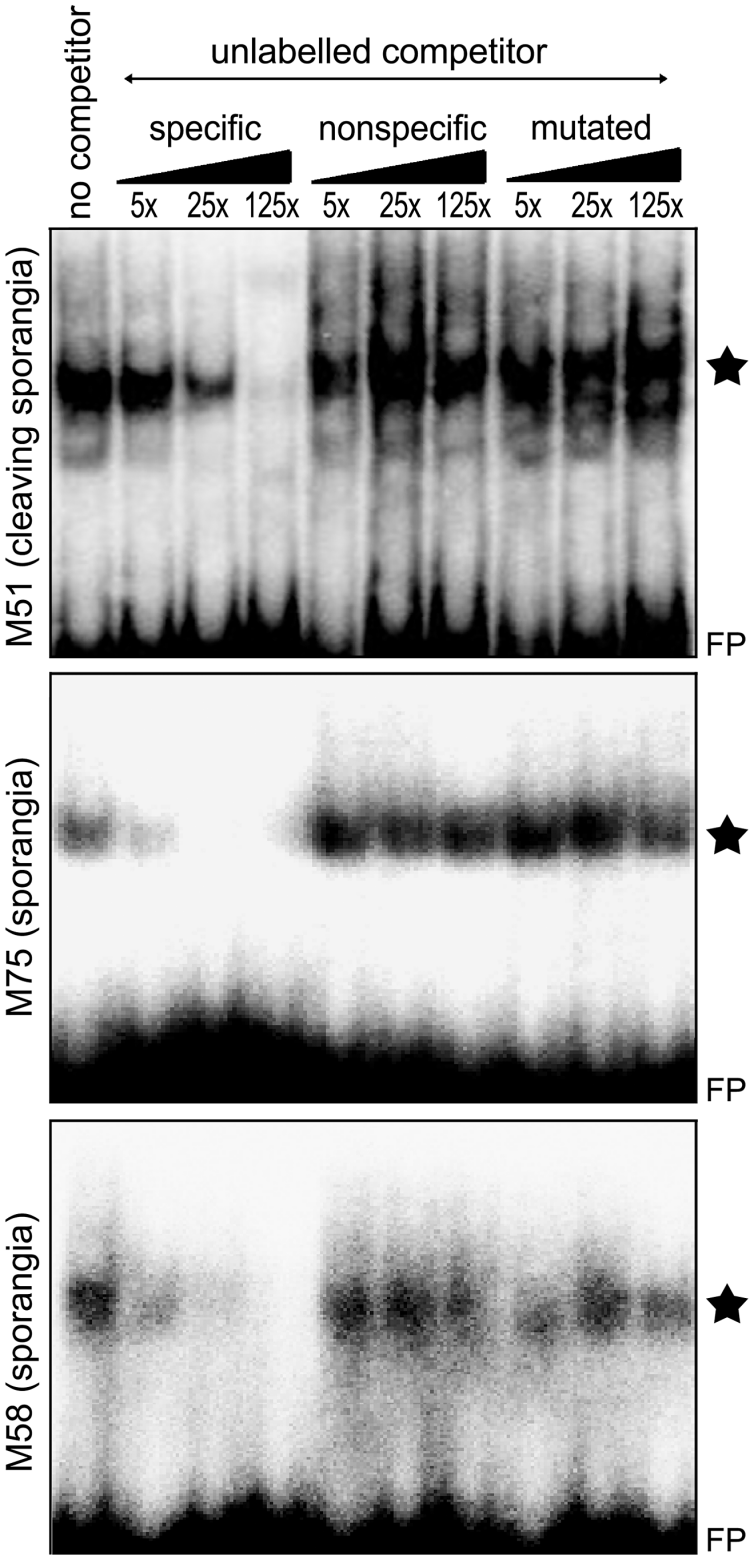
Electrophoretic mobility shift assays. Nuclear proteins from cleaving sporangia or sporangia were mixed with ^32^P-labeled double-stranded oligonucleotides containing M51, M75, and M58, and subjected to electrophoresis. Reactions were performed either with no competitor, or unlabeled competitors at 5×, 25×, and 125× the concentration of labeled probe. These were either a specific competitor (same as labeled probe), a nonspecific competitor containing unrelated sequences, or mutated competitors derived from the specific competitor but mutated for the motif. Stars indicate the specific bands, and FP stands for free probe.

Further support for the motifs was provided by electrophoretic mobility shift assays (EMSA) involving M51, M58, and M75. As shown in Figure 10, each motif bound a protein from nuclear extracts of cleaving sporangia (M51) or sporangia (M58, M75). Binding appeared to be specific based on comparing different unlabeled competitors. These included a specific competitor (same sequence as the labeled probe), a nonspecific competitor (a random sequence), and mutated competitor (same as the labeled probe, but with the motif mutated). In each case the nonspecific and mutated competitors had little effect in reducing the binding of the labeled probe, compared to the specific competitor. For M51, several bands were detected, which was suggestive of a multi-protein complex or the binding of proteins of different sizes.

### Association of motifs with pathogenicity factors

Several classes of proteins have been identified that play roles in pathogenesis, of which many are secreted and sometimes induced during infection [29], [36], [37]. To assess the usefulness of our data for understanding how such genes are regulated, we checked their promoters for the motifs, focusing on motifs associated with the germinating cyst/appressoria stage. This involved analyzing the main classes of genes annotated by Raffaele et al. [29] as potentially encoding secreted pathogenicity factors, of which many are induced during plant infection. As shown in Table 1, four motifs associated with the germinating cyst/appressoria stage and ten linked to both the hyphal and germinating cyst/appressoria stages were over-represented (*p*<0.05) in such genes. These included those encoding plant cell-wall degrading enzymes, glucanase inhibitors, Nep1-like (NLP) toxins, PcF toxins, elicitins and elicitin-like proteins (potential sterol carriers), proteases, protease inhibitors, and RXLR effectors. As expected, motifs linked to stages such as sporangia and zoospores were typically under-represented (Table S2).

**Table 1:** Motifs associated with early infection stages in promoters of genes encoding secreted pathogenesis candidate proteins.

	Name of over-represented motif (number of genes)	
Protein group	GC-associated	GC and HY-associated
Cell wall degrading enzymes (cutinase, polygalacturonase, pectin esterase, pectate lyase)	M19	M87, M93
Crinklers	none	M93
Glucanase inhibitors	M103	M4, M80, M87, M93
Nep1-like (NLP) proteins	M19, M95	M4, M12, M13, M87, M89, M93
PcF toxins	M101	M4, M12, M87, M93, M94
Potential sterol carriers (elicitin and elicitin-like)	M95	M4, M87, M89, M93, M94
Proteases	M19, M95	M80
Protease inhibitors	M95, M103	M4, M87, M89, M92, M93
RXLR effectors	M95, M101, M103	M4, M80, M87, M89, M92, M93, M94, M96

Gene groupings employed the annotations of Raffaele et al. [29]. The table reports motifs associated with the germinating cyst with appressoria (GC) or the GC and hyphal (HY) stages that were over-represented in each group at *P*<0.05; the complete data are in Table S2.

Not all genes in each group contained a germinated cyst/appressoria motif in their promoters, however. For example, such motifs were in only 225 of the 493 RXLR promoters, with 66 containing M95, 163 having M101, and 49 having M103 (Table S2). As only some RXLR genes are induced during infection [18], [29], [38], we checked for a correlation between motif and expression pattern. RXLR genes with a germinated cyst/appressoria motif were more likely to be infection-induced than those without; many had more than one motif, with a correlation between the degree of induction and motif number (*r* = 0.27, *p* = 0.04). Crinkler genes, which are not typically infection-induced but are considered to encode pathogenicity factors due to the ability of some to produce necrosis in plants [18], had M93 as the sole over-represented motif. M93, a palindrome, is over-represented in both germinated cyst/appressoria and hyphal-induced genes and occurs 4150 times within *P. infestans* promoters. Its abundance suggests that it is associated with general growth and not specifically with pathogenesis.

### Predictive value of motifs

We assessed the extent to which the presence of a motif predicts a gene's expression pattern. This involved searching promoters of all 7,862 genes on the microarrays for motifs associated with sporangia-induced genes and germinated cyst/appressoria genes, using 500-nt of DNA upstream of the start codon as the search space. We then compared motif frequencies in promoters induced by >5-fold at each stage versus non-induced promoters. The 99 sporangia-induced and 103 germinated cyst/appressoria-induced promoters used originally for motif discovery were excluded from these analyses, to test if our earlier results extended to all *P. infestans* genes.

Each of the 11 sporangia-associated motifs occurred more often in the induced promoters than non-induced controls (Table 2). On average, each motif was 66% more likely to occur in an induced promoter, with individual motifs showing a 21 to 100% enrichment. For example, M8 was found in 14.3% of induced promoters compared to 11.6% of non-induced promoters, representing a 23% enrichment. It is important to note that hits due to random chance are always expected to greatly exceed the number of functional TFBSs for reasons elaborated upon in Discussion [39].

**Table 2:** Frequency of motifs in up-regulated promoters.

		Occurrence of motif		
Motif and associated stage		% of induced genes (I)	% of non-induced genes (NI)	Ratio (I/NI)
Sporangia	M8	14.3	11.6	1.23
	M10	2.0	1.0	2.00
	M29	0.6	0.4	1.60
	M32	6.1	3.9	1.57
	M43	12.3	7.3	1.69
	M45	3.7	2.8	1.35
	M64	6.6	4.5	1.45
	M65	6.1	4.6	1.33
	M68	3.7	1.9	1.99
	M82	3.9	3.1	1.26
	M90	2.0	1.7	1.21
Germinated	M4	29.2	20.8	1.40
cysts with	M13	13.5	12.6	1.07
appressoria	M14	1.3	1.4	0.93
	M15	1.3	1.4	0.93
	M26	1.3	1.1	1.18
	M48	1.4	1.2	1.17
	M80	5.2	4.1	1.27
	M85	1.5	1.2	1.25
	M87	18.0	12.1	1.49
	M88	14.1	11.5	1.23
	M89	11.0	9.3	1.18
	M92	1.9	1.5	1.27
	M93	19.3	13.9	1.39
	M95	3.8	3.8	1.00
	M96	12.0	12.2	0.98

Values are percent of genes containing the indicated motifs within 500-nt of the start codon. For the two classes of motifs, induced genes (I) are defined as having 5-fold or higher mRNA levels in sporangia relative to hyphae, or in germinated cysts relative to zoospores. Non-induced genes were those having mRNA levels that did not change or were down-regulated during the transition. Motifs were not included if they were over-represented in constitutive promoters.

Most of the 15 motifs linked to the germinated cyst/appressoria stage were also over-represented in that stage when the total microarray data were analyzed (Table 2). Each was on average 19% more likely to be in an induced promoter compared to controls. It is notable that four of the motifs were not enriched in the genome-wide set of induced promoters, including M95. Since our functional tests showed that M95 conferred expression in the germinated cyst/appressoria stage, it is possible that M95 binds a bifunctional transcription factor or has its activity mediated by other transcription factors.

We also checked for the association of a sporulation-associated motif with expression pattern in ten genes from *P. infestans* that were not on the microarrays. Motif M8 was chosen for this exercise simply since it was first on the list in Table 2. We identified promoters containing M8, used RT-qPCR to measure mRNA in sporangia and nonsporulating hyphae, and assessed if M8 was within the orthologous promoter from *P. sojae* (Table 3). Of six *P. infestans* genes in which the *P. sojae* ortholog also contained the motif, five were induced by >2-fold in sporangia. This was significant (*p* = 0.004), compared to the likelihood of this fraction of genes being induced by random chance. In contrast, none of the four *P. infestans* genes that lacked M8 in their *P. sojae* ortholog was induced based on the 2-fold cutoff.

**Table 3:** Expression pattern of genes containing M8.

	Motif locations (within 600 nt 5′ of translation start)		
*P. infestans* gene	*P. infestans*	*P. sojae* ortholog	mRNA ratio (sporangia/hyphae)
PITG_02417	−137	−192	24.5
PITG_02250	−224	−230	6.8
PITG_03691	−112	−131	6.5
PITG_02149	−128	−88	6.1
PITG_02224	−356	−167	3.8
PITG_02027	−289	−301	1.9
PITG_02337	−226	none	1.6
PITG_02430	−337	none	1.4
PITG_02048	−499	none	0.9
PITG_02729	−203	none	0.9

## Discussion

Genome-wide searches for promoter motifs shared by co-expressed genes have been performed in model animals, plants, and fungi [14]–[17], but only on a limited scale in pathogens [40], [41]. The strategy seemed attractive for *Phytophthora* since its modest transformation efficiencies make motif discovery through traditional means challenging [42]. The success of our approach was shown not only by the identification of 100 putative TFBSs, but the fact that all six motifs tested performed as predicted in functional assays. Nearly all motifs also exhibit at least one feature typical of TFBSs besides over-representation such as positional bias, orientation bias, or evolutionary conservation. Discovering the motifs, which include several associated with pathogenicity factors, is a key step towards understanding the networks that control development and host infection in *Phytophthora* and similar approaches should be useful in other pathogens.

Several features contributed to our approach by increasing the sensitivity of our searches and reducing false positives. First, our requirement that motifs be identified by two of three algorithms served as a stringent filter. Second, we focused on promoters that show large changes, which was possible since major shifts in mRNA levels occur during the *P. infestans* life cycle as about 12% of genes change by >100-fold in the stages addressed by this study [23]. Third, gene models were manually curated to accurately define the search space. Finally, since intergenic distances are typically small in *P. infestans*, most regulatory regions were probably within the 1-kb search space.

Our analysis of the overall transcriptional landscape of *P. infestans* has also helped illuminate the structure and function of its promoters; few promoter studies have previously been performed in the entire Kingdom Stramenopila, which includes diatoms and brown algae in addition to oomycetes [43]. Remarkably, the median intergenic distance within gene-dense regions of *P. infestans* is even less than that of most yeasts [25], [44]. The ratio of adjacent *P. infestans* genes that are transcribed in the same direction versus from a shared or adjacent promoter region is 1.43, which is higher than that of *S. cerevisiae* and *A. thaliana* [45]. This presumably reflects functional constraints associated with having small adjacent promoters, which is reflected in the co-expression or anti-correlated profiles of about 10% of adjacent *P. infestans* genes. Excluding cases where co-expressed pairs are duplicated genes, most adjacent genes in *P. infestans* are nevertheless transcribed independently. Our prediction of directionality for more than half of the motifs helps to explain how this independence is mediated. The predominant mechanism for regulating transcription in *Phytophthora* may also not involve chromatin-level effects, which in yeasts and metazoans are inferred to extend up to 4 kb and tens of kilobases, respectively [46]. Nevertheless, of the approximately 300 transcription factors annotated within each *Phytophthora* genome, several belong to families associated with chromatin remodeling [47].

Relative simplicity in transcriptional regulation in *P. infestans* is also implied by our finding that each of six stage-induced motifs tested conferred tissue-specific expression with a minimal promoter. Combinatorial control, not counting transcription factor heterodimerization, thus may not be a principal feature of stage-specific regulation in oomycetes, unlike other eukaryotes with complex genomes [48], [49]. Since position effects in *P. infestans* make it challenging to compare transgene expression between transformants [50], our data are silent on roles of other TFBSs in quantitative expression. The potential involvement of only a few TFBSs per gene is consistent with our observation of limited blocks of similarity between *P. infestans*, *P. ramorum* and *P. sojae* promoters, as shown in Figure 6. As the three organisms are relatively distant in molecular phylogenies [51] and have significant morphological differences, it would be useful to know if the orthologs had similar patterns of expression.

Our analyses of motifs associated with sporangia and germinated cyst/appressoria stages (Table 2) suggests that the occurrence of a motif has utility in predicting expression pattern. However, it is important to recognize the limitations of this approach. Since TFBSs are short and often degenerate, they occur by random chance in great abundance. Moreover, TFBS function depends on chromatin structure and often the co-occurrence of other TFBSs. Some transcription factors are also bifunctional, leading to different outcomes depending on post-translational modification or co-regulators [52]. Because of such complications, Wasserman and Sandelin [39] posited the “futility theorem” which states that the great majority of predicted TFBSs lack function: it is thus futile to predict expression based on the occurrence of a promoter motif. Our experience in *P. infestans* was more encouraging, however. For example, while the presence of a sporulation-associated motif was a weak forecaster of expression pattern, the predictive value was fairly strong if the motif was conserved in another *Phytophthora.* The limits of predictions based solely on motif presence can be illustrated for the 11 sporulation-associated motifs in Table 2. Based on their average size and base composition, and using 500-nt of promoter sequences as a search space, about 8250 promoters should contain one or more of the 11 motifs by random chance. Extrapolating from microarray data, however, only about 2200 genes are sporulation-induced, so random hits exceed functional TFBSs by a 4 to 1 ratio. Nevertheless, a future can be envisioned where better predictions of expression based on motif occurrence alone may be possible. In *S. cerevisiae*, a network model that integrated expression patterns of 2,587 genes under 255 conditions of growth and development with 666 TFBS definitions using AND, OR, and NOT logic resulted in fairly good predictions of expression of about 3/4 of the genes [53], [54].

Inferences about the complexity of the networks that control development and pathogenesis in *P. infestans* may be drawn from our observation that roughly 10 to 20 motifs were linked to each stage of the life cycle. This is consistent with observations that show that sporangia and zoospore formation involves several steps and signaling pathways [20], [55], [56]. Characterizing transcription factors that bind the motifs will help reveal details of these pathways, and enable chromatin immunoprecipitation studies to confirm the target genes [57]. Studying the transcription factors may also lead to strategies for blocking diseases, by interfering with the expression of proteins used for overcoming host barriers and defenses.

## Materials and Methods

### Microarray and promoter datasets for *P. infestans*

Expression data were from a prior study that used Affymetrix microarrays to measure mRNA during the stages addressed by this paper [23]. Reliable expression calls were detected for 12,463 of the 15,650 sequences targeted by the arrays. Since the microarrays predated the current draft genome which is based on strain T30-4 (available from the Broad Institute of Harvard and MIT), we linked the microarray sequences to annotated T30-4 genes using BLASTN, but excluded genes on small contigs to reduce errors in analyses of intergenic distances and co-expression. By selecting the best hit with >97% identity, 7,862 genes with reliable expression data in the five life-stages were matched, including 3944 adjacent gene pairs of which 2937 were within 2 kb of each other. These were mapped to the assembly, omitting unexpressed or missing genes.

Datasets of *P. infestans* promoters included 1-kb of sequences 5′ of predicted open reading frames. Total promoters were downloaded from the Broad Institute database, and then subsets were extracted using custom scripts. Sets included promoters from the differentially-expressed gene sets described above, in which mRNA levels were induced by at least 7.5-fold compared to the prior development stage (*p*<0.05 based on replicates). Sets of at least 100 promoters were used for calculating 5′ intergenic distances. For identifying over-represented motifs, analyses were limited to genes induced >10-fold, which corresponded to 99, 95, 46, 103, and 100 in the sporangia, cleavage, zoospore, germinated cyst, and hyphal sets, respectively. Prior to extracting promoters, gene models were examined and corrected as needed (changing 8, 14, 17, 3, and 9 promoters, respectively). This mostly involved eliminating introns that contradicted EST evidence, or spanned regions that when converted to exons maintained the reading frame and had high similarity to *P. ramorum* and *P. sojae* orthologs. In addition, a constitutive promoter dataset was established from 150 genes that showed <25% variation between the stages. Promoters from *P. ramorum* and *P. sojae* were extracted from genome assemblies downloaded from the Virginia Bioinformatics Institute.

### Algorithms for detecting over-represented motifs

Stand-alone versions of MEME (version 4.3.0; [12]), YMF (version 3.0; [9]), and BioProspector (release 2; [10]) were employed. MEME ran with minimum and maximum widths of 5 and 8, respectively, using 5 iterations. Gap opening and extension costs were 11 and 1, respectively, any number of repetitions were allowed, and the E-value cut-off was 10^−5^. YMF used a value of 8 for lenOligo (the number of non-spacer characters) and output was sorted by *z*-score. BioProspector used a value of 8 for motif width, with the 100 top motifs reported per run. The program was run 10 times on each set of promoters and a PERL script was used to eliminate redundant motifs.

Initial outputs (382 motifs from BioProspector from the five stage-induced promoter datasets, 450 from MEME, and 1261 from YMF) were submitted to a PERL script to detect motifs detected by at least two programs. These were then merged to eliminate redundancy, allowing degeneracy at two sites. *P* values for over-representation of the final motifs were calculated based on a hypergeometric distribution, using Fisher's Exact test. The locations, numbers, and orientations of each motif were extracted from the datasets using custom Perl scripts, with tests for orientation bias employing Chi square. Motifs that were positionally biased were identified by counting the number of hits per 200-nt bin, extending 1-kb upstream of the start codon, and checking for deviations from a random allocation model using a 2 by 5 Fisher's Exact test.

### Tests for evolutionary conservation

Candidate orthologs were identified in *P. capsici*, *P. ramorum*, and *P. sojae* genome databases (from the Joint Genome Institute of the U. S. Department of Energy) using BLASTP. Their promoters were then extracted, and aligned with CLUSTALW using gap opening and extension penalties of 10 and 0.1, respectively, and DIALIGN using default parameters [58], [59]. After preliminary tests, *P. capsici* was omitted since the version of its genome assembly available at the time contained too many gaps and erroneous gene models. Putative three-way orthologs (*P. infestans*, *P. ramorum*, *P. sojae*) were identified for 66% of genes. Up to five genes (mean = 4.6) were typically examined for each motif. If a motif appeared in the alignment at the same position in at least one comparison it was scored as being evolutionarily conserved. A score of “ambiguous” was given for motifs found in a different location (including by searching in both orientations); on average, 8% of promoters would have a false positive. Conservation at the same site in ortholog sets for all genes was never expected: gene models in the different species often started at different locations, errors may have occurred in selecting orthologs, not all orthologs might have similar expression patterns, and promoter rearrangements are common during evolution.

### Manipulations of *P. infestans*

Stable transformants were generated from isolate 1306 (from tomato in California, USA) using a liposome-assisted protoplast method as described [42], except that Extralyse (Laffort, Bordeaux, France) was used as the β-glucanase. Non-sporulating mycelia were obtained by inoculating clarified rye-sucrose broth with a sporangial suspension (10^4^/ml), followed by 48 hr incubation at 18°C. Sporangia were obtained from rye-sucrose agar cultures by adding water, rubbing with a glass rod, and passing the fluid through a 50 µm mesh to remove hyphal fragments. To induce cleavage, sporangia were placed in 100 mm glass culture dishes resting on ice (internal temperature 8–10°C) for 60 min. Germinated cysts were obtained by allowing the chilled sporangia to release zoospores, to which CaCl_2_ was added to 0.5 mM followed by vortexing for 1 minute and incubation at 18°C for up to 9 hr. Gene expression analyses involved RNA blotting and β-glucuronidase (GUS) staining as described [21].

Constructs for testing promoters were based on pNPGUS, which is an improved version of pOGUS [60], and pNIFS-NPGUS. Each contains a promoterless GUS gene and an *nptII* selectable marker driven by the *ham34* promoter. The improvements in pNPGUS included the addition of additional cloning sites upstream of GUS (the polylinker from pBS-KS2+) and translational stop codons upstream of the polylinker to reduce the number of cryptic transcripts with GUS activity. pNIFS-NPGUS contains a 74-nt minimal promoter from the *NifS* gene of *P. infestans* [21], [61] cloned into *Xma*I and *Eco*RI sites of the polylinker. Promoter fragments were inserted into pNPGUS or pNIFS-NPGUS as fragments amplified by polymerase chain reaction, or by ligating double-stranded oligonucleotides into the *Xba*I and *Xma*I sites of the vectors. Oligonucleotides used for cloning are listed in Table S3.

### Electrophoretic mobility shift assays (EMSA)

Nuclear protein isolation and EMSA were as described [22], except that heparin agarose was not used for the extractions. EMSA involved mixing 5 µg of nuclear protein with 1 µg poly dI-dC and 1.6 ng of ^32^P-labeled probe in buffer containing 1 mM dithiothreitol for 15 min at room temperature followed by 30 min on ice, followed by electrophoresis at room temperature on a 4.5% acrylamide gel. For competition assays, protein was incubated with unlabeled DNA for 15 min and then the labeled probe for 30 min on ice. Double-stranded oligonucleotides generated using the sequences in Table S3 were used as probe and cold (unlabeled) competitors. Mutated competitors were altered for the predicted motifs (A↔C, G↔T), and the nonspecific probe was a random sequence.

### Quantitative RT-PCR

qRT-PCR employed DNAse-treated RNA, pooled from two biological replicates, which was reverse-transcribed using oligo-dT with a first-strand synthesis kit from Invitrogen (Carlsbad, CA, USA). Amplifications employed hot-start *Taq* polymerase with primers targeted to the 3′ regions of genes, typically yielding amplicons of 100 to 125 nt, using SYBR Green as a reporter. Reactions were performed in duplicate using the following conditions: one cycle of 95°C for 8 min, and 35 cycles of 95°C for 20 s, 55°C for 20 s, and 72°C for 30 s. Controls lacking reverse transcriptase and melting curves were used to test the data. Results were normalized based on primers for a constitutively expressed gene encoding ribosomal protein S3a, and expression was determined by the ΔΔCT method.

## Supporting Information

Figure S1 Distribution of genes with different expression patterns within *P. infestans* supercontigs. Color-coding is the same as in Figure 2.
(TIF)

Table S1 Details of promoter motifs.
(XLS)

Table S2 Occurrence of motifs in promoters of potential secreted pathogenicity genes.
(XLS)

Table S3 Oligonucleotides employed in this study.
(PDF)
