# Supplementary material for: Genome-wide Prediction and Functional Validation of Promoter Motifs Regulating Gene Expression in Spore and Infection Stages of Phytophthora infestans
Source: PLoS Pathog. 2013 Mar 14;9(3):e1003182. doi: 10.1371/journal.ppat.1003182 (PMC3597505; doi:10.1371/journal.ppat.1003182)
Supplement: Table S3 — Oligonucleotides employed in this study. (PDF) [file ppat.1003182.s004.pdf]

Table S3. Oligonucleotides employed in this study.

Name	Sequence (5'-3')	Purpose
C38.2U	CTAAAATAGATAAGGCGGCCGCTCTAGAACTAGTGGATC CCCCGGGCTGCAGGAATTCAATAAAAT	Add polylinker to pOGUS (upper strand)
C38.2L	CGATTTTATTGAATTCCTGCAGCCCGGGGGATCCACTAG TTCTAGAGCGGCCGCCTTATCTATTTTAGGGCC	Add polylinker to pOGUS (lower strand)
NifSU	TCCCCCCGGGGGATTGAAGATTCGACGG	Amplify *NIFS* minimal promoter for insertion in pNPGUS, upper primer
NifSL	GGAATTCCCGTTGTAGCCGTGGT	Amplify *NIFS* minimal promoter for insertion in pNPGUS, lower primer
4671U	GCTCTAGAAGAAACTGAGCCTCGGTATGA	Amplify 500 bp promoter from PITG_16321
4671L	CGGAATTCAGAAATGCTAAGCGAAGACTG	Amplify promoter from PITG_16321 (lower primer for all constructs)
Del1U	GCTCTAGAGCCGTCGGTATCCAAGAGGTA	Amplify 312 bp promoter from PITG_16321
Del2U	GCTCTAGAGCCTCCCTGCTGTCGTCCTC	Amplify 187 bp promoter from PITG_16321
Del3U	GCTCTAGAGCGGGTCCGTCTTCTAGTCCA	Amplify 104 bp promoter from PITG_16321
Mut1U	GCTCTAGAGCAGAAACCTCACCGTCCTCGAACCACA	Amplify 187 bp promoter from PITG_16321 with LB mutation, pMUT1
Mut2U	GCTCTAGAGCCTCCCTGCTGTACAATCCGAACCACATGG CAT	Amplify 187 bp promoter from PITG_16321 with M51 mutation, pMUT2
Mut2U	GCTCTAGAGCCTCCCTGCTGTCGTCCTCGTTGTTGTATTT ATGTGCTCCCATCCG	Amplify 187 bp promoter from PITG_16321 with RB mutation, pMUT3
BLKU	CTAGACTCCCTGCTGTCGTCCTCGAACCACATGGCTTCC CGTCTTCTCGTCTC	Annealed with BLKL to form LB-M51-RB region in pOLIGO1
BLKL	CCGGGAGACGAGAAGACGGGAAGCCATGTGGTTCGAGG ACGACAGCAGGGAGT	Annealed with BLKU to form LB-M51-RB region in pOLIGO1
OC-U	CTAGACGTCCTCGGGTTGGTGCAATTTCCCGTCTTCTCG TCTC	Annealed with OC-U to form M51 region (with 37 random 3' bases) in pOLIGO2

OC-L	CCGGGAGACGAGAAGACGGGAAATTGCACCAACCCGAG GACGT	Annealed with OC-U to form M51 region from PITG_16321 in pOLIGO2
SP2_OC-U	CTAGACTTCAACGAGTTGGTGCAATTTCCCGTCTTCTCGT CTACGTCCC	Annealed with SP2_OC-L to form M58 region for NifS-GUS fusion
SP2_OC-L	CCGGGGGACGTAGACGAGAAGACGGGAAATTGCACCAA CTCGTTGAAGT	Annealed with SP2_OC-U to form M58 region for NifS-GUS fusion
SP1_OC_U	CTAGAGCTGCTGCGGTTGGTGATTTCCCGTCTTCTCG TCTACGTC	Annealed with SP2_OC-L to form M75 region for NifS-GUS fusion
SP1_OC_L	CCGGGGGACGTAGACGAGAAGAGGGAAATTGCACCAAC CGCAGCAGT	Annealed with SP2_OC-U to form M75 region for NifS-GUS fusion
GC1_OC_U	CTAGAGTACATGTAGTTGGTGCAATTTCCCGTCTTCTCGT CTACGTCCC	Annealed with SP2_OC-L to form M93 region for NifS-GUS fusion
GC1_OC_L	CCGGGGGACGTAGACGAGAAGAGGGAAATTGCACCAAC TACATGTACT	Annealed with SP2_OC-U to form M93 region for NifS-GUS fusion
GC2_OC_U	CTAGACTATTAATAGTTGGTGCAATTTCCCGTCTTCTCGT CTACGTCCC	Annealed with SP2_OC-L to form M95 region for NifS-GUS fusion
GC2_OC_L	CCGGGGGACGTAGACGAGAAGAGGGAAATTGCACCAAC TATTAATAGT	Annealed with SP2_OC-U to form M95 region for NifS-GUS fusion
MK_1_U	CTAGAGGAAGAAGGTTGGTGCAATTTCCCGTCTTCTCGT CTACGTCCC	Annealed with MK_1_L to form M64 region for NifS-GUS fusion
MK_1_L	CCGGGGGACGTAGACGAGAAGACGGGAAATTGCACCAA CCTTCTTGCT	Annealed with MK_1_U to form M64 region for NifS-GUS fusion
MK_3_U	CTAGAGCAACAACGTTGGTGCAATTTCCCGTCTTCTCGTC TACGTCCC	Annealed with MK_1_L to form M95 region for NifS-GUS fusion
MK_3_L	CCGGGGGACGTAGACGAGAAGACGGGAAATTGCACCAA CGTTGTTGCT	Annealed with MK_1_U to form M95 region for NifS-GUS fusion
EMSA_CL_SPEC_UP	CTCCTAGACTCCCTGCTGTCGTCCTCGAACCACATGGCT CCCGTCT	Specific probe for M51 motif, upper strand
EMSA_CL_SPEC_LO	AGACGGGAGCCATGTGGTTCGAGGACGACAGCAGGGAG TCTAGGAG	Specific probe for M51 motif, lower strand
EMSA_CL_NS_UP	TCGAGTACTTCTACACCATCATGGCACTGTACTCCTCTAG TCTGTA	Non-specific probe for all motifs, upper
EMSA_CL_	TACAGACTAGAGGAGTACAGTGCCATGATGGTGTAGAAG	Non-specific probe for all motifs, lower

NS_LO	TACTCGA	
EMSA_CL_MUT_UP	CTCCTAGACTCCCTGCTGTACAATCCGAACCACATGGCTCCCGTCT	Mutated probe for M51 motif, upper
EMSA_CL_MUT_LO	AGACGGGAGCCATGTGGTTCGGATTGTACAGCAGGGAGTCTAGGAG	Mutated probe for M51 motif, lower
EMSA_SP2_SPEC_UP	CTGCCTCCTCCAATTTGCACTTCAACTTGTGTAGCCATCTGACGACC	Specific probe for M58 motif, upper
EMSA_SP2_SPEC_LO	GGTCGTCAGATGGCTACACAAGTTGAAGTGCAAATTGGAGGAGGCAG	Specific probe for M58 motif, lower
EMSA_SP2_Mut_UP	CTGCCTCCTCCAATTTGCAAGGTCAATTGTGTAGCCATCTGACGACC	Mutated probe for M58 motif, upper
EMSA_SP2_Mut_LO	GGTCGTCAGATGGCTACACAATTGACCTTGCAAATTGGAGGAGGCAG	Mutated probe for M58 motif, lower
EMSA_SP1_SPEC_UP	AGCGAGTCGATGTCTCCGCCGCTGCTGTGCACGTCACCAACCAGGGA	Specific probe for M75 motif, upper
EMSA_SP1_SPEC_LO	TCCCTGGTTGGTGACGTGCACAGCAGCGGCGGAGACATCGACTCGCT	Specific probe for M75 motif, lower
EMSA_SP1_MUT _UP	AGCGAGTCGATGTCTCCGCCAAGCTATTGCACGTCACCAACCAGGGA	Mutated probe for M75 motif, upper
EMSA_SP1_MUT_LO	TCCCTGGTTGGTGACGTGCAATAGCTTGGCGGAGACATCGACTCGCT	Mutated probe for M75 motif, lower
